# The complete mitochondrial genome sequences of *Bupleurum falcatum* (Apiales: Apiaceae)

**DOI:** 10.1080/23802359.2020.1781566

**Published:** 2020-06-24

**Authors:** Chang-Kug Kim, Min-Woo Jin, Yong-Kab Kim

**Affiliations:** a Genomics Division, National Institute of Agricultural Sciences, Jeonju, Korea; b School of Information Communication Engineering, Wonkwang University, Iksan, Korea

**Keywords:** Apiaceae family, *Bupleurum falcatum*, *Daucus carota*, mitochondrial genome

## Abstract

*Bupleurum falcatum* has a long history of use in traditional oriental medicine. The first complete mitochondrial genome sequences of *B. falcatum* were 463,792 bp based on 494,582 aligned reads. A total of 51 genes was annotated including 32 protein-coding genes, 16 tRNA genes, and three rRNA genes. In a comparison of *B. falcatum* and carrot (*Daucus carota*) revealed that the former species has four exclusive genes, but lacks six genes present in the latter. The compositional structure and phylogenetic relationships indicated that the mitochondrial genome of *B. falcatum* is similar to that of *D. carota*.

*Bupleurum falcatum*, a member of family Apiaceae, has a long history of use in traditional oriental medicine. The plant has immunomodulatory, anti-inflammatory, antiviral, anti-tumorigenesis, and anti-ulcer activities (Ashour and Wink 2011; Lee et al. 2012). A sample was obtained from the National Agrobiodiversity Center (voucher number: IT258576, geographic coordinate: 35°49′19′′N, 127°8′56′′E) in Jeonju, Korea. Whole-genome shotgun sequencing was performed by the Illumina sequencing platform (Illumina, San Diego, CA, USA). First, paired-end (PE) libraries were constructed with an insert size of 270–700 bp, and raw reads with Phred scores ≤30 were removed using the CLC-quality trim tool of CLC Package 4.06 (CLC Inc., Aarhus, Denmark). Second, remaining reads were assembled using the CLC de novo assembly tool, and chloroplast-derived sequences of *B. falcatum* (Shin et al. 2016) were used as a reference. The complete mitochondrion of B. falcatum (Accession no. KX887330) was registered to NCBI GenBank.

The complete mitochondrial genome of *B. falcatum* was a circular form of 463,792 bp in length using 494,582 aligned reads with average coverage of 90.5 depth. The genome harbored 51 annotated genes, including 32 protein-coding genes, 16 tRNA genes, and three rRNA genes. The annotated protein-coding genes mostly encode well-known proteins such as electron transport chain (Hofmann 2013), rRNAs are commonly used to elucidate evolutionary relationships between organisms (Smit et al. 2007), and tRNAs are indispensable components of protein translation (Novoa et al. 2012). In a comparison of the same Apiaceae family, *B. falcatum* and carrot (*Daucus carota*), their mitochondrial genomes differ significantly in size (463 kb vs. 281 kb) and gene order. However, number of unique genes was similar (51 vs. 53), and total of 47 genes with highly conserved sequences were common to both genomes. Comparison of the mitochondrial genome structures of *B. falcatum* and *D. carota* revealed that the former species has four exclusive genes (i.e. *rps10*, *rps14*, *tatC*, and *trnX*), but lacks six genes (i.e. *mttb*, *rpl2*, *rpl10*, *rps7*, *rps13*, and *trnfM-CAU*) present in the latter. The phylogeney of B. falcatum was reported using genetic markers and ribosomal DNA (Neves and Watson 2004); however, mitochondrial phylogeny using the whole-genome sequence in B. falcatum has not yet been reported. Phylogenetic tree was constructed using MEGA X (Kumar et al. 2018) with 12 common protein-coding genes (i.e. *atp4, atp6, ccmB, ccmC, cob, cox1, cox3, nad2, nad3, nad9, rps3*, and *rps12*) in the 10 reported species. The phylogeny indicated that *B. falcatum* species is most closely related to the *D. carota* of Apiaceae family, and *B. falcatum* is definitely separated from the Fabaceae family (Figure 1). This study will contribute to develop molecular markers for germplasm classification and breeding of new varieties of Apiaceae.

**Figure 1.**
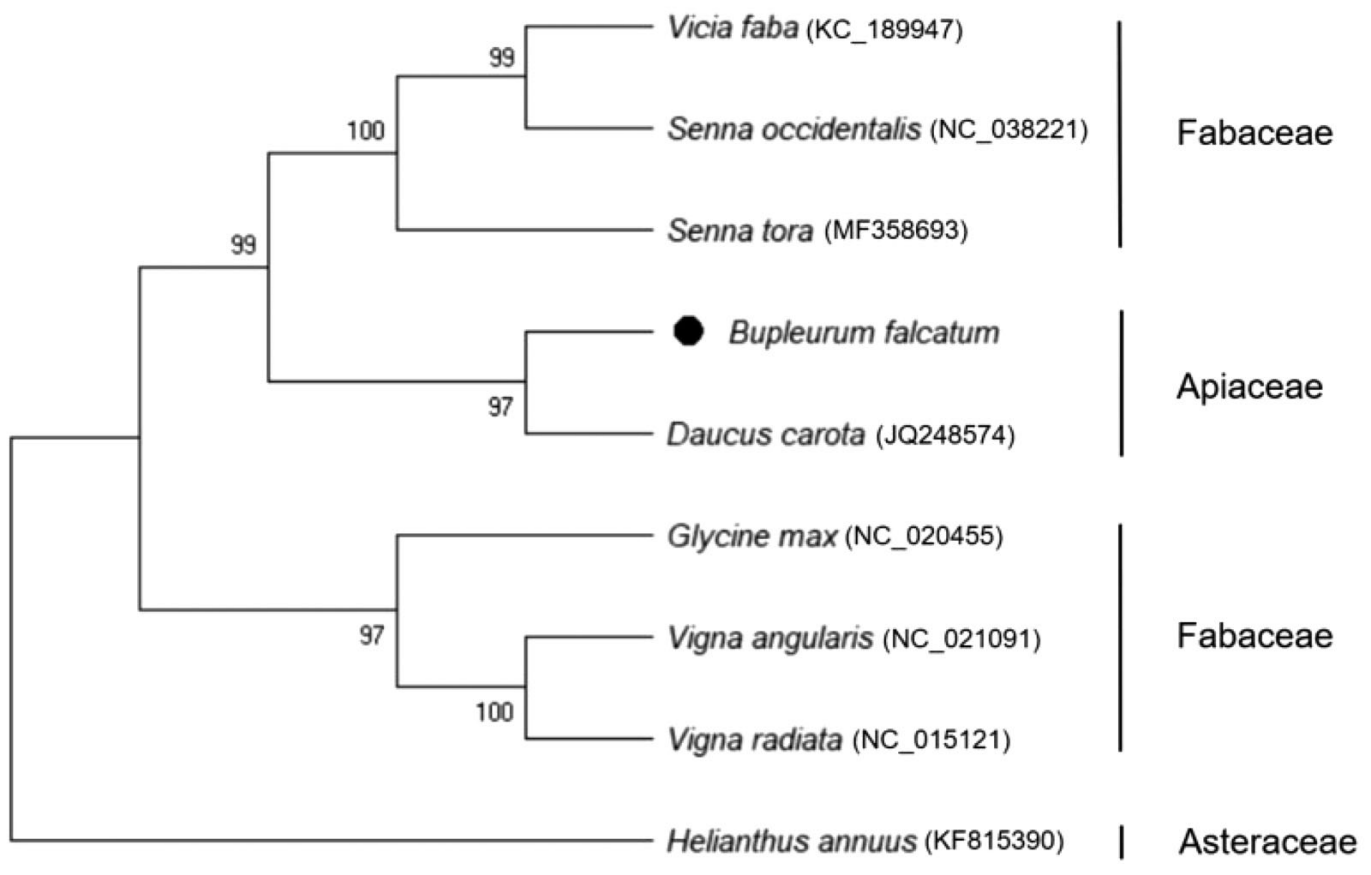
Phylogeny of *B. falcatum* and nine related species based on mitochondrial genome sequences. The phylogenetic tree was constructed using the Maximum-Likelihood method and JTT matrix-based model based on 12 common protein-coding genes of nine species mitochondrial genomes.

## Geolocation

The genomic DNA sample was obtained from the National Agrobiodiversity Center (voucher number: IT258576, geographic coordinate: 35°49′19′′N,127°8′56′′E) in Jeonju, Korea.

## Data Availability

The data that newly obtained at this study are available in the NCBI under accession number of KX887330 (https://www.ncbi.nlm.nih.gov/nuccore/KX887330.1/).
